# Regulation of the autophagy system during chronic contractile activity‐induced muscle adaptations

**DOI:** 10.14814/phy2.13307

**Published:** 2017-07-18

**Authors:** Yuho Kim, David A. Hood

**Affiliations:** ^1^ Muscle Health Research Centre School of Kinesiology and Health Science York University Toronto Ontario Canada; ^2^ School of Kinesiology and Health Science York University Toronto Ontario Canada

**Keywords:** Autophagy, exercise, lysosome, mitochondrial biogenesis

## Abstract

Skeletal muscle is adaptable to exercise stimuli via the upregulation of mitochondrial biogenesis, and recent studies have suggested that autophagy also plays a role in exercise‐induced muscle adaptations. However, it is still obscure how muscle regulates autophagy over the time course of training adaptations. This study examined the expression of autophagic proteins in skeletal muscle of rats exposed to chronic contractile activity (CCA; 6 h/day, 9V, 10 Hz continuous, 0.1 msec pulse duration) for 1, 3, and 7 days (*n *= 8/group). CCA‐induced mitochondrial adaptations were observed by day 7, as shown by the increase in mitochondrial proteins (PGC‐1α, COX I, and COX IV), as well as COX activity. Notably, the ratio of LC3 II/LC3 I, an indicator of autophagy, decreased by day 7 largely due to a significant increase in LC3 I. The autophagic induction marker p62 was elevated on day 3 and returned to basal levels by day 7, suggesting a time‐dependent increase in autophagic flux. The lysosomal system was upregulated early, prior to changes in mitochondrial proteins, as represented by increases in lysosomal system markers LAMP1, LAMP2A, and MCOLN1 as early as by day 1, as well as TFEB, a primary regulator of lysosomal biogenesis and autophagy flux. Our findings suggest that, in response to chronic exercise, autophagy is upregulated concomitant with mitochondrial adaptations. Notably, our data reveal the surprising adaptive plasticity of the lysosome in response to chronic contractile activity which enhances muscle health by providing cells with a greater capacity for macromolecular and organelle turnover.

## Introduction

The skeletal muscle adaptations that occur in response to endurance exercise training result in increased aerobic performance. This is attained via an upregulation of mitochondrial biogenesis and function (Hood 2001). In addition to training‐induced mitochondrial adaptations, recent work has suggested that autophagy is also an important player in skeletal muscle remodeling (Lira et al. 2013; Vainshtein et al. 2015a,b; Memme et al. 2016), whereby damaged and unnecessary cellular components are degraded and recycled, contributing to the maintenance or improvement of muscle quality following chronic exercise.

Autophagy can be initiated by nonselectively sequestering cytosolic substrates within a double‐membrane structure, a phagophore, which is conjugated with ubiquitination‐linked proteins. These include both p62 and the lipidated form of microtubule‐associated protein light chain II (LC3 II) (Pankiv et al. 2007). After maturing into the autophagosome, the double‐membrane vesicle is fused to the lysosome, wherein the engulfed substrates are degraded by lysosomal proteases such as cathepsin D. Autophagy can also selectively engulf mitochondria, a process called mitophagy. Damaged and dysfunctional mitochondria accumulate PINK1 (PTEN‐induced kinase (1) on the outer membrane (Youle et al. 2015). This serves to recruit Parkin, an E3 ubiquitin ligase, which ubiquitinates outer membrane proteins, thereby serving to “tag” mitochondria for degradation via mitophagy (Youle et al. 2015). In addition, autophagy can occur without constructing autophagosomes, in which autophagic substrates can be directly delivered to the lysosome through the interaction with a chaperone protein such as heat shock cognate protein 70 (HSC70) (Massey et al. 2006; Kaushik et al. 2008). This substrate‐HSC70 complex can be selectively recognized by a lysosomal receptor (i.e., LAMP2A) and then degraded in the lysosomal lumen, via the process of chaperone‐mediated autophagy (CMA) (Massey et al. 2006; Kaushik et al. 2008). Collectively, autophagy can be accomplished by coordinating these different mechanisms, all of which contribute to degrade cellular waste products.

Within the last decade, a growing number of studies have investigated the effects of exercise on these autophagy systems in skeletal muscle of humans and rodents. Those studies have adopted either acute or chronic exercise regimens in the experimental design, but the time course of adaptive changes in autophagy has so far remained unresolved. In addition, the lysosomal system is essential for the regulation of autophagy, and this is primarily dependent on the activity of transcription factor EB (TFEB) (Settembre et al. 2011; Spampanato et al. 2013). In particular, TFEB regulates not only lysosomal biogenesis, but also autophagosomal degradation, thus governing an overall balance of the lysosomal system. However, only few studies have been conducted to identify a role of TFEB in skeletal muscle exposed to changes in muscular activity (Vainshtein et al. 2015b), and more work is required to investigate the exercise‐ and/or training‐induced effects on the lysosomal system. We hypothesized that contractile activity‐induced mitochondrial adaptations would be matched with concomitantly regulated autophagy and lysosomal systems that would support muscle mitochondrial quality control, and we investigated how these degradation systems are altered during the chronology of skeletal muscle adaptations.

## Methods

### Animals and chronic contractile activity

Male Sprague‐Dawley rats (468 ± 8 g, *n* = 24) were purchased from Charles River Laboratories (St. Constant, QC, Canada) and were given food and water ad libitum. After at least 5 days as an adaptive period, all animals were surgically processed to undergo chronic contractile activity (CCA), in accordance with the guidelines set by the York University Animal Care Committee. CCA surgeries were performed as done previously (Connor et al. 1985; Adhihetty et al. 2007; Memme et al. 2016) under anesthesia with isoflurane, and small incisions were made to allow a sterile wire electrode (Medwire, Leico industries, New York, NY) to pass subcutaneously from the top of the back to the left hindlimb. The electrodes were sutured on both sides of peroneal nerve that innervates the tibialis anterior (TA) and extensor digitorum longus (EDL) muscles. The other ends of electrodes were connected to an external stimulator (9V, 10 Hz continuous, 0.1‐msec pulse duration, 999 msec of rest per second), which was fixed with surgical tape, and designed to be activated with infrared light. The contralateral hindlimb was used as the control muscle. After 5–7 days of recovery, animals were subjected to CCA stimulation for 6 hours per day for 1, 3, or 7 days, respectively (*n* = 8 per group). All animals were then killed to collect TA and EDL muscles 17–18 h after the last CCA stimulation. All collected tissues were snap‐frozen in liquid nitrogen and stored at −80°C until further biochemical analysis.

### COX activity

Cytochrome c oxidase (COX) enzyme activity was used to assess mitochondrial content. Pulverized whole TA muscle was diluted in an extraction buffer (100 mmol/L Na‐K‐Phosphate, 2 mmol/L EDTA, pH 7.2) and sonicated three times at 30% power output on ice. Using a Synergy‐HT microplate reader (Biotek Instruments, Winooski, VT), COX activity was determined by reading the rate of change in cytochrome c oxidation at 550 nm, as done previously (Vainshtein et al. 2015a,b; Memme et al. 2016).

### Whole muscle extraction and western blotting

Pulverized whole TA muscles were suspended in extraction buffer (20 mmol/L HEPES, 2 mmol/L EGTA, 1% Trion X‐100, 50% glycerol, 50 mmol/L *β*‐Glycerophosphate) and were rotated for 1 h at 4°C. The muscle tissues were sonicated three times for 3 sec at 30% power output and then centrifuged at 16,000*g* for 10 min at 4°C. Supernates were collected and protein concentrations were determined using the Bradford assay. Proteins (20~30 *μ*g) were separated by SDS‐PAGE and transferred onto nitrocellulose membranes (120V, 2 h). The membranes were blocked with 5% milk buffer for 1 h at room temperature, and they were incubated with individual primary antibodies at 4°C overnight. Antibody information is provided in Table 1. On the following day, corresponding secondary antibodies were incubated for 1 hour at room temperature. Using chemiluminescent substrates (Clarity ECL Western blotting substrates, Bio‐Rad, CA), antibodies bound to target proteins were developed, and they were analyzed using ImageJ software (Version 1.48, NIH, USA).

**Table 1:** List of antibodies

Antibody	Manufacturer	Reference No.	Lot No.
PGC‐1*α*	Millipore	AB3242	2691399
COX I	Abcam	ab14705	GR233531‐3
COX IV	Abcam	ab140643	GR192963‐3
LC3 I/II	Cell Signaling	4108	3
p62	Sigma	P0067	015M4877V
ATG7	Sigma	A2856	110M4861
Beclin‐1	Cell Signaling	3738	3
PINK1	Santa Cruz Biotechnologies	SC‐33796	C2107
Parkin	Cell Signaling	4211	4
TFEB	MyBioSource Inc.	MBS120432	—
LAMP1	Abcam	ab24170	GR268500‐1
Cathepsin D	Santa Cruz Biotechnologies	SC‐6486	J1111
HSC70	Abcam	ab2788	GR255118‐5
LAMP2A	Life Technologies	512200	QD216942
MCOLN1 (Mucolipin1)	Abcam	ab28508	GR281984‐1
GAPDH	Abcam	ab8245	GR137268‐5
*β*‐actin	Santa Cruz Biotechnologies	SC‐47778	A1416

### Single fiber immunofluorescence

As shown in our previous study (Vainshtein et al. 2015a) and by others (Raben et al. 2009), fresh EDL fibers were fixed and immunofluorescent signals against lysosomal markers were obtained using confocal microscopy. Briefly, fresh EDL muscles were fixed with 2% paraformaldehyde for at least 1 h. The fixed muscle fibers were stored in 50% glycerol at 4°C overnight and then kept at −20°C until further analysis. After gradually decreasing the concentration of glycerol, the muscles were gently teased apart in a 0.04% saponin solution and incubated in blocking solution (0.2% Triton X‐100, 10% goat serum in PBS) for 1 h at room temperature. The fibers were then coincubated with primary antibodies against LAMP1 (Abcam, ab24170; 1:200) and Cathepsin D (Santa Cruz, sc‐6486; 1:100) at 4°C overnight. After washing with PBS, the fibers were coincubated with corresponding secondary antibodies (Goat anti‐rabbit IgG H&L [Alexa Fluor^®^ 488, ab150077; 1:200] and donkey anti‐goat IgG H&L [Alexa Fluor^®^ 647, ab150131; 1:200]) conjugated with Alexa fluor^®^ for immunofluorescence for 2 h at room temperature. Next, the tissues were washed three times with PBS, and DAPI (0.5 *μ*g/mL) was added during the first wash. The muscle fibers were then mounted on glass slides with mounting solution (ProLong^®^ Diamond Antifade Mountant, P36961, Life Technologies, OR). Using a Zeiss confocal microscope (LSM700, Urbana, IL), confocal immunofluorescent images were obtained using a ×40 objective and the Zen software program (Zeiss, Urbana, IL).

### Statistical analysis

All data were analyzed using Prism software 7.0 (GraphPad, San Diego, CA) and are shown as Mean ± SEM. We performed two‐way ANOVA to determine the statistical significance of the main effects and the interaction (CCA × Time). When statistical significance was found, the Bonferroni's post hoc test was performed to identify significant individual comparisons. For correlation analyses, Pearson's analysis was conducted. Statistical significance was set as *P* < 0.05.

## Results

### CCA induces mitochondrial adaptations in skeletal muscle

**Figure 1:**
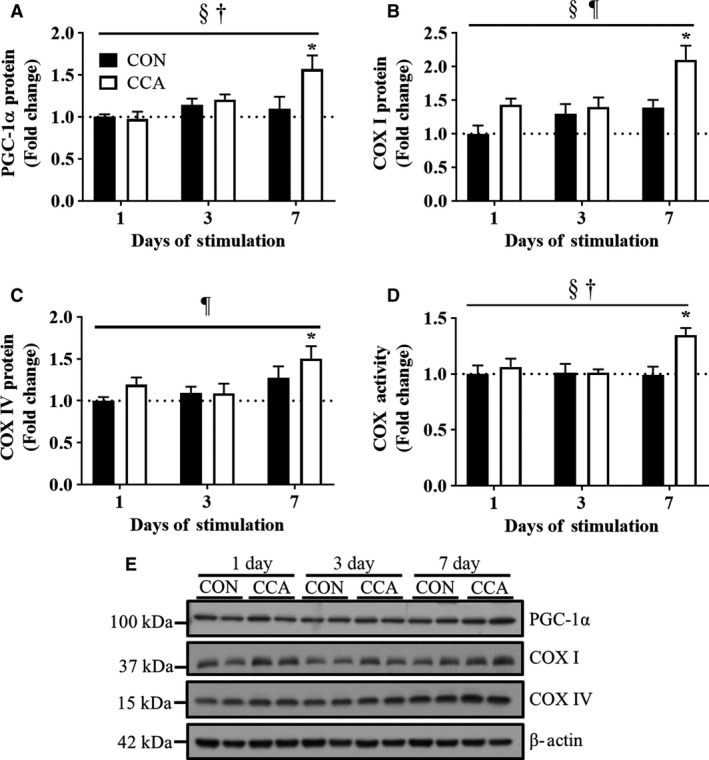
Chronic contractile activity (CCA) induces mitochondrial adaptations in skeletal muscle by day 7. Fold‐change in the protein expression of mitochondrial biogenesis markers (A) PGC‐1*α*, (B) COX I, and (C) COX IV (*n* = 8 per group). (D) Fold‐change in the COX activity for mitochondrial contents (*n* = 6 per group). (E) Representative blots for PGC‐1*α*, COX I, and COX IV. *β*‐Actin was used as loading control. Molecular weights on the left of blots are for protein ladders. All bars are indicative of fold‐change relative to the control group at Day 1, and they represent means ± SEM. †*P* < 0.05, interaction effect between CCA and time; §*P *< 0.05, main effect of CCA; ¶*P* < 0.05, main effect of time; *P < 0.05, significant difference versus control at Day 1.

As in our previous studies (Adhihetty et al. 2007; O'Leary and Hood 2008; Memme et al. 2016), we have successfully shown that 7 days of electrical stimulation‐induced CCA led to mitochondrial adaptations in rat skeletal muscle. As expected, CCA increased the protein level of PGC‐1*α* by 1.6‐fold at day 7 (Fig. 1A, 1E), compared to basal levels (*P *< 0.05). To further verify mitochondrial adaptations in this model, we also tested protein levels of both mitochondrial‐ and nuclear‐encoded COX I and COX IV which are cytochrome c oxidase subunits in the electron transport chain (Fig. 1B, C and E). The protein abundances of both COX I and COX IV were increased after each time point, which culminated at day 7 as compared to day 1. Further, we also observed that CCA increased COX I protein levels over the entire CCA experimental procedure. In addition, COX holoenzyme activity was significantly higher by 1.3‐fold at day 7 following CCA compared with contralateral, nonstimulated muscle (*P *< 0.05; Fig. 1D).

### CCA induces adaptations in autophagy protein expression

**Figure 2:**
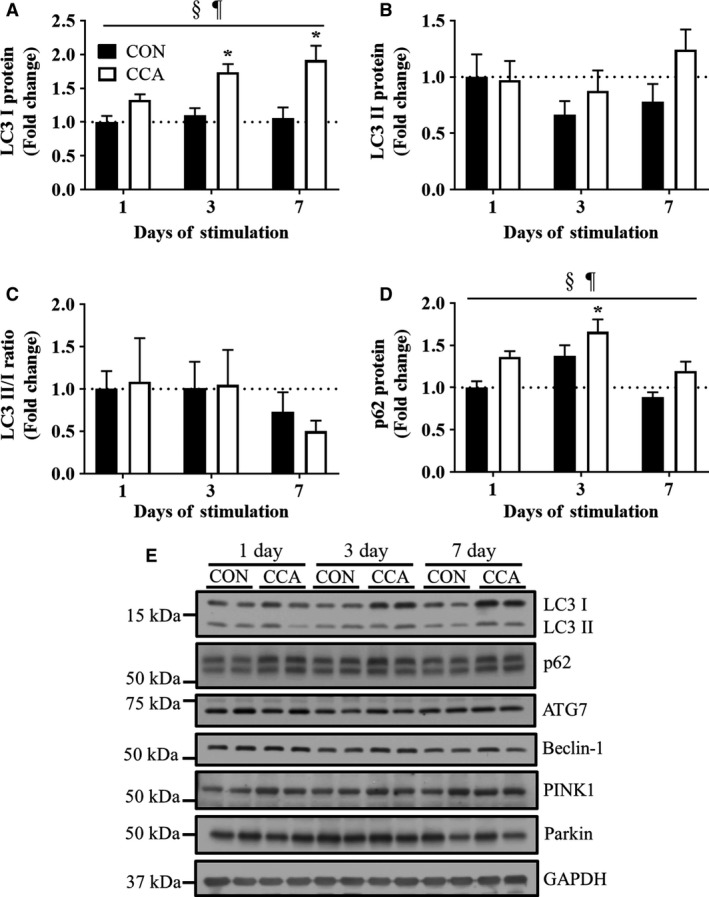
Autophagy protein changes during CCA‐induced muscle adaptations. Fold‐change in the protein levels of autophagy induction markers (A) LC I, (B) LC II, (C) LC3 II/LC3 I, and (D) p62 (*n* = 8 per group). Both the ratio of LC3 II to LC3 I and p62 were used for an indicator of autophagy flux that may reflect autophagosomal degradation. (E) Representative blots for LC3 I/II, p62, ATG7, Beclin‐1, PINK1, and Parkin. GAPDH was used for loading control. All bars are shown as fold‐change relative to the control group at Day 1, and they represent means ± SEM. §*P *< 0.05, main effect of CCA; ¶*P* < 0.05, main effect of time; *P < 0.05, significant difference versus control at Day 1.

To examine how the autophagy system is regulated in skeletal muscle in response to CCA, we targeted the adaptor proteins, LC3 (II and I) and p62, as well as upstream regulatory proteins Beclin‐1 and ATG7. LC3 I protein levels were gradually increased by CCA throughout 7 days, reaching ~1.9‐fold higher values compared with basal levels (*P *< 0.05; Fig. 2A). Unlike LC3 I, we detected no change in protein levels of LC3 II, a lipidated‐active form of LC3 I. The ratio of LC3 II to LC3 I was calculated to estimate autophagy flux. Although the ratio was maintained constant by day 3 regardless of CCA treatment, CCA tended to reduce this ratio (*P* = 0.07) by ~50% at day 7 compared to the basal levels at day 1.

p62 is an adaptor protein that conjugates autophagy substrates with LC3 II, which triggers autophagosomal formation. p62 protein levels were significantly elevated by CCA as early as day 3 (Fig. 2D), but then decreased toward basal levels at day 7 (*P *< 0.05). Along with the decreased ratio of LC3 II/I, this reduction from an elevated level following 7 days of CCA suggests an increased autophagy flux during progressive skeletal muscle adaptations.

To better understand these LC3 and p62 results, we also examined the autophagy upstream protein markers, ATG7 and Beclin‐1. Our result revealed that neither ATG7 nor Beclin‐1 was significantly affected by CCA over the time course (graphs not shown, *P* > 0.05). Similarly, we also sought to evaluate how CCA altered the expression of the important mitophagy markers Parkin and PINK1 in whole cell lysates of skeletal muscle. In this study, we failed to detect any significant difference in the level of these proteins as a result of up to 7 days of CCA (graphs not shown, *P* > 0.05; Fig. 2E).

### The lysosomal system is increased prior to CCA‐induced mitochondrial adaptations

**Figure 3:**
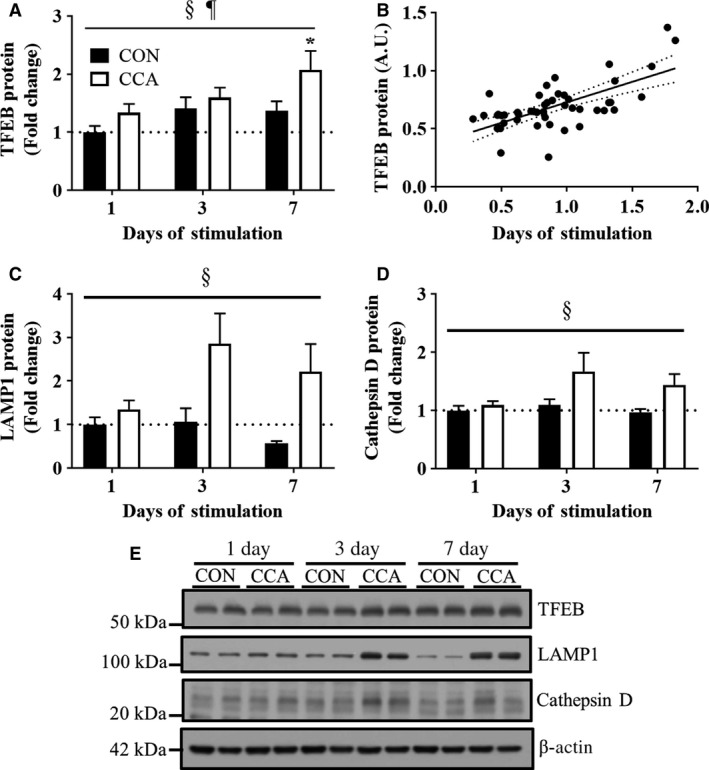
Lysosomal protein alterations during muscle adaptations. Fold‐change in the protein expression of lysosomal system markers (A) TFEB, (C) LAMP1, and (D) Cathepsin D (*n* = 8 per group). The protein levels in Cathepsin D show only the mature forms (~33 kDa). (B) Pearson's correlation between TFEB and PGC‐1*α* proteins (*n* = 48 per protein; *P* < 0.05). (E) Representative blots for TFEB, LAMP1, and Cathepsin D. *β*‐actin was used for loading control. All bars are shown as fold‐change relative to the control group at Day 1, and they represent means ± SEM. §*P *< 0.05, main effect of CCA; ¶*P* < 0.05, main effect of time; *P < 0.05, significant difference versus control at Day 1.

Since the lysosome is a key organelle for the maintenance of the autophagy system, we also sought to understand how the lysosomal system is regulated by CCA. TFEB, a master regulator of lysosomal biogenesis, was shown to be elevated by CCA at all time points (*P *< 0.05; Fig. 3A). TFEB levels were also significantly correlated with those of PGC‐1*α* (*r* ^*2*^ **= 0.4375, *P *< 0.05; Fig. 3B), suggesting that TFEB and PGC‐1*α* may coregulate each other in skeletal muscle.

**Figure 4:**
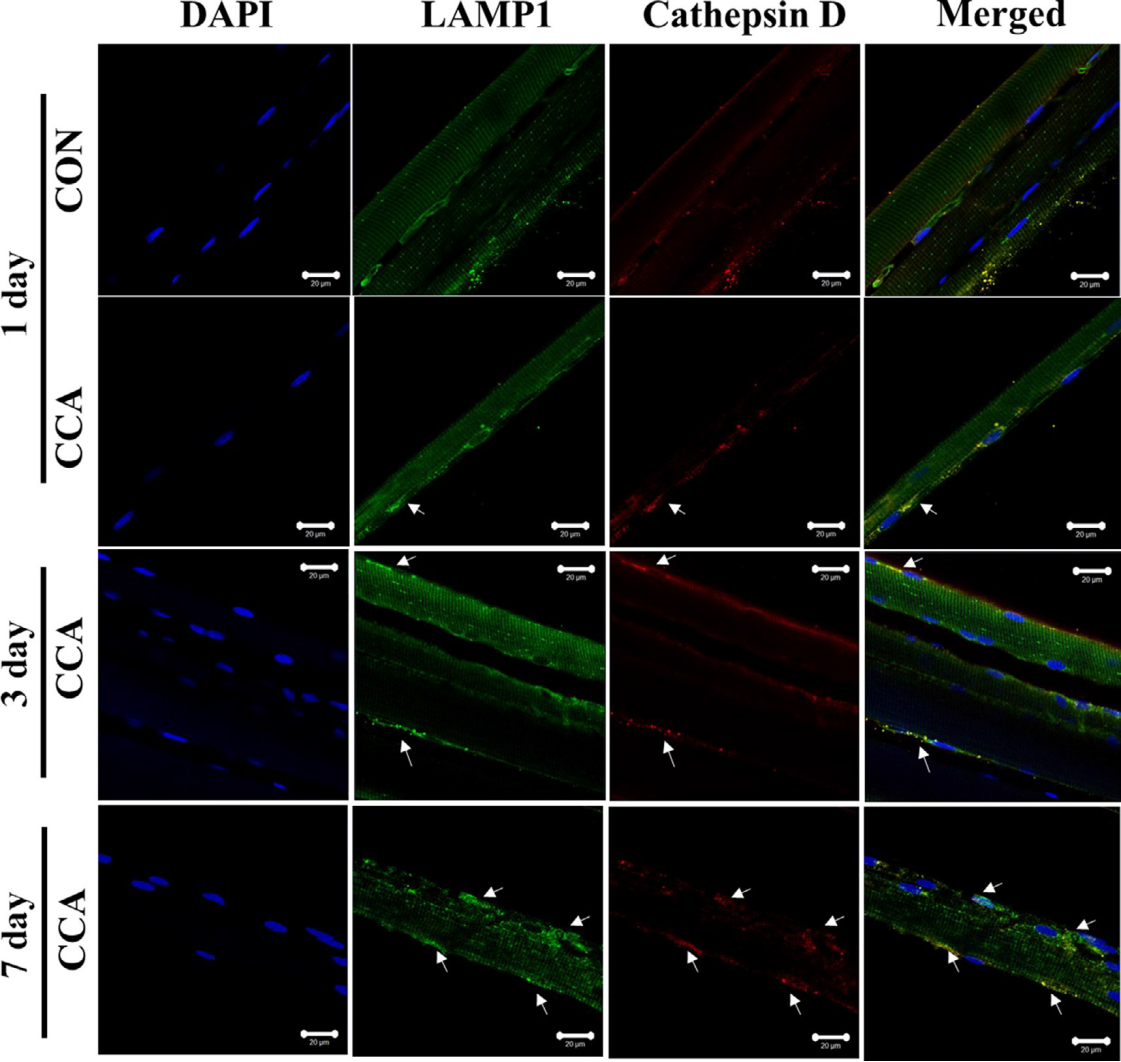
Confocal images of fixed single fibers immunostained for lysosomal markers. Confocal images for immunofluorescent LAMP1 (green) and cathepsin D (red) are obtained from the fixed EDL single fibers, and Merge images (yellow) show colocalization of LAMP1 and cathepsin D. DAPI images (blue) display the location of nuclei. Arrows indicate prominent expression of target proteins. Scale bar indicates 20 *μ*m.

In addition to TFEB, we also examined lysosomal markers LAMP1 and cathepsin D, which are a lysosomal receptor and protease, respectively. CCA markedly increased the protein levels of LAMP1 and the active form of Cathepsin D (i.e., 33 kDa) over the course of CCA (*P* < 0.05). We also visualized the colocalization of those lysosomal markers to better understand CCA‐induced regulations of the lysosomal system using confocal microscopy. Corroborating the data shown in the western blots, the immunofluorescent colocalizations of LAMP1 and Cathepsin D were more prominent following 3 and 7 days of CCA (Fig. 4). Collectively, these results suggest that CCA induces a marked increase in lysosomal biogenesis as an early event in response to an exercise stimulus in skeletal muscle.

### CMA is induced during CCA

**Figure 5:**
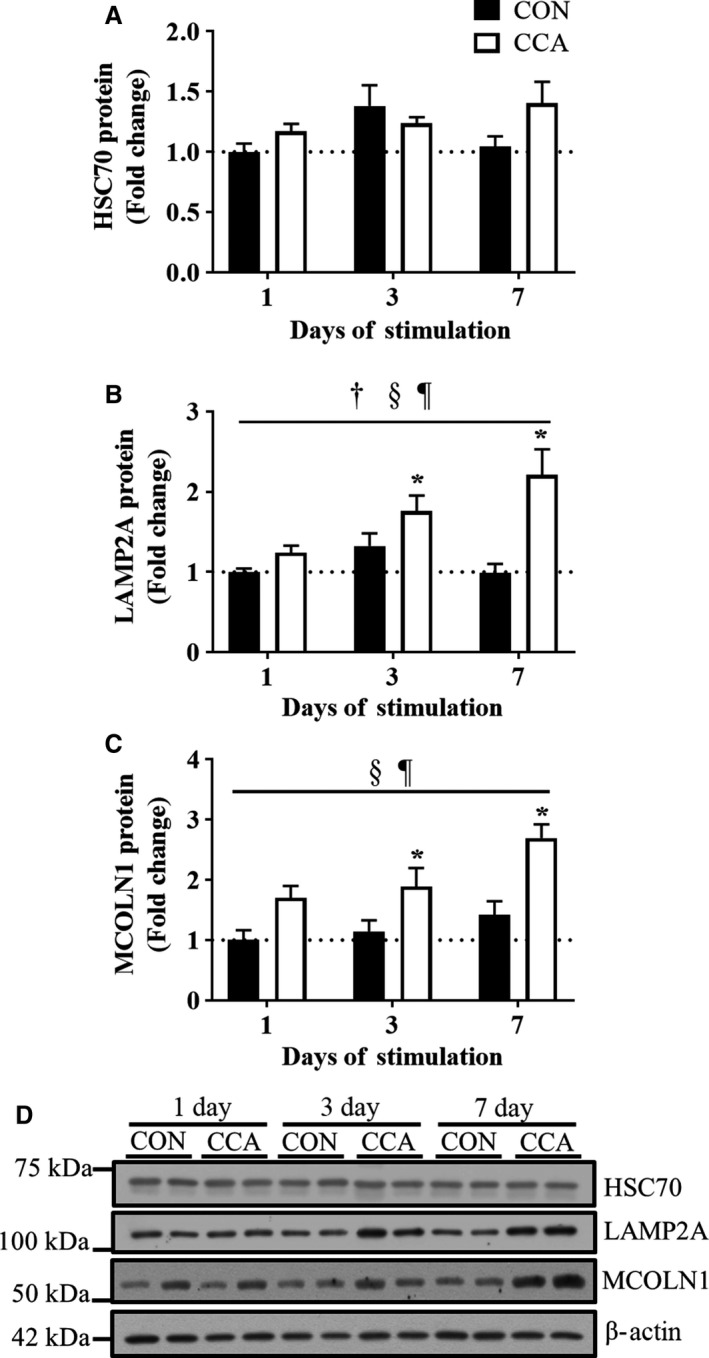
CCA induces chaperon‐mediated autophagy protein expression in skeletal muscle. Fold‐change in the protein expression of chaperone‐mediated autophagy (CMA) markers (A) HSC70, (B) LAMP2A, and (C) MCOLN1 (*n* = 8 per group). (D) Representative blots for HSC70, LAMP2A, and MCOLN1. *β*‐actin was used for loading control. All bars are shown as fold‐change relative to the control group at Day 1, and they represent means ± SEM. †*P* < 0.05, interaction effect between CCA and time; §*P *< 0.05, main effect of CCA; ¶*P* < 0.05, main effect of time; *P < 0.05, significant difference versus control at Day 1.

We also examined how CCA regulates the CMA system in skeletal muscle. First, we evaluated the protein abundance of HSC70, a constitutively expressed chaperone directly carrying autophagic substrates to the lysosome. While CCA had no apparent effect on the levels of HSC70, a notable ~2.5 fold‐increase in LAMP2A, a lysosomal receptor for HSC70‐bound substrates, was observed by 7 days (*P *< 0.05; Fig. 5B). In addition, we detected a significant effect of CCA on the protein accumulation of mucolipin 1 (MCOLN1), a lysosomal calcium channel (*P* < 0.05; Fig. 5C). CCA was shown to increase MCOLN1 protein levels at all time points, and the extents appeared to be significant by day 3 and 7, respectively (*P *< 0.05). Thus, these data suggest that lysosomal adaptations represent a relatively early event in the muscle remodeling to chronic exercise.

## Discussion

Skeletal muscle is characterized by plasticity in which its functional and structural character can be regulated to meet various metabolic demands. Mitochondrial biogenesis and autophagy are established to be key pathways in the cellular remodeling of skeletal muscle. However, it is not known if these systems are coregulated during training adaptations. We hypothesized that there would be some coordination of these two processes because muscle mitochondrial quality is determined by a balance of both biogenesis and mitophagy, the specific autophagic degradation of mitochondria. Hence, we sought to understand how these two skeletal muscle remodeling systems are regulated over 7 days of chronic contractile activity (CCA), a well‐defined experimental model for endurance exercise (Takahashi et al. 1998; Ljubicic et al. 2004; Adhihetty et al. 2007; O'Leary and Hood 2008; Memme et al. 2016). Our findings provide evidence that the autophagy and lysosomal systems adapt prior to, or concomitant with, mitochondrial changes in skeletal muscle exposed to chronic contractile activity.

As shown in the previous studies (Ljubicic et al. 2004; Adhihetty et al. 2007; Memme et al. 2016), we observed that PGC‐1*α*, a master regulator of mitochondrial biogenesis, was upregulated by CCA in skeletal muscle by day 7, which was supported by the increased whole muscle COX activity. Although COX I and COX IV protein levels were changed in a time‐dependent manner, they showed similar protein expression patterns with PGC‐1*α*, suggesting the likelihood of concurrent CCA‐induced mitochondrial biogenesis. Collectively, these findings indicate that mitochondrial biogenesis is significantly induced by day 7 in this CCA model.

We sought to understand how skeletal muscle autophagy systems are altered by CCA at multiple time points (day 1, 3, and 7). Our results indicate that while CCA had a significant effect in increasing LC3 I protein abundance over 7 days, LC3 II levels remained unchanged by the CCA treatment. In addition, our current data, along with the earlier findings (Memme et al. 2016), indicate that the ratio of LC3 II to LC3 I decreased between days 3 and 7. Our previous study also revealed that LC3 I mRNA was unaffected by up to 7 days of CCA (Memme et al. 2016). Taken together, these data suggest that LC3 I transcription is not enhanced, but rather that the protein is stabilized within muscle at the onset of CCA‐induced adaptations. LC3 I seemed to be not immediately converted to LC3 II, which implies a limiting step in the lipidation process. Also, the lack of a coordinated change in ATG7 levels in response to CCA, a component of the complex responsible for LC3 I lipidation to LC3 II, supports this. Nonetheless, the tendency for a reduced LC3 II to LC3 I ratio between days 3 and 7 suggests that muscle autophagic flux may become more activated when the CCA‐induced mitochondrial adaptations culminate. Likewise, other studies have also claimed that endurance exercise training decreased the LC3 II to LC3 I ratio in the skeletal muscle of humans (Fritzen et al. 2016) and rodents (McMillan et al. 2015), probably due to the increase in LC3 I protein abundance.

Changes in the levels of p62 are often used as a surrogate marker of autophagy flux, because p62 is degraded via the autophagy pathway. Other studies have found that p62 levels in muscle are either not changed (Greene et al. 2015; McMillan et al. 2015; Nilsson et al. 2015), downregulated (Lira et al. 2013), or increased (Fritzen et al. 2016) in response to endurance exercise training. These differential results in p62 may be attributable to the use of various exercise protocols (chronic vs. intermittent muscle activity), species, muscle fiber types, or the time of sampling during the adaptive process (Lira et al. 2013). Our results using the CCA model show that p62 protein levels peaked at an early time point (i.e., day 3) and then returned back to basal levels over the time course of muscle adaptations. We have also found that this is preceded by increases in p62 mRNA (Memme et al. 2016), suggesting an increased transcriptional drive for p62 synthesis at the onset of muscle adaptations. Based on these results, we conjecture that muscle likely reprograms autophagy flux to a higher level following the training‐inducible adaptations. An interesting observation was that p62 protein levels in the contralateral muscle were also increased by CCA, suggesting a possible cross‐over effect not evident with other protein adaptations. Similarly, Fritzen et al. (2016) reported that one‐legged endurance exercise increased p62 protein abundance in both exercised and contralateral nonexercised leg. Further studies are clearly required to determine the reason for this, as well as to more accurately determine CCA‐associated change in muscle autophagic flux, through administration of agents such as colchicine that are known to block autophagosomal degradation (Ju et al. 2010, 2016; Vainshtein et al. 2015b).

Unlike autophagy induction makers, we failed to detect any significant changes in other autophagy‐related proteins (i.e., ATG7 and Beclin‐1) over the time course of training adaptations. Likewise, others have also reported similar results in their exercise studies, showing that short‐ (Smuder et al. 2011) and long‐ (Lira et al. 2013; McMillan et al. 2015; Tam et al. 2015) term endurance training do not alter those transcript and/or protein levels in skeletal muscle. Instead, these autophagy markers seem to be more susceptible to acute endurance exercise rather than chronic exercise protocols, even though mixed results have been reported (Kim et al. 2012; Vainshtein et al. 2015b). For example, Vainshtein et al. (2015b) revealed increased skeletal muscle protein levels of ATG7 and Beclin‐1 after a bout of treadmill exercise, however opposite results were found by another group (Kim et al. 2012).

We observed no significant effect of CCA on markers of mitochondria‐specific autophagy (i.e., mitophagy), reflected in the lack of change in Parkin and PINK1 protein expression. Similarly, other groups have also identified comparable results to our finding, showing that gene and/or protein levels of Parkin and PINK1 are not altered by acute (Jamart et al. 2012) or chronic (Ju et al. 2016) exercise. However, all of these studies, including ours, have been limited to determining mitophagy using whole muscle cell lysates, not mitochondrial fractions. Our previous work suggested an effect of acute running on the upregulation of mRNA levels, as well as Parkin localization on mitochondrial fractions of mouse skeletal muscle (Vainshtein et al. 2015b). However, more research with mitochondrial‐specific samples is warranted to better assess the muscle mitophagy system following chronic exercise, in comparison to mitochondrial biogenesis.

The lysosome is an integral part in regulating the autophagy system, which is mainly altered by TFEB, a primary transcription factor for lysosomal biogenesis and integrity. However, to the best of our knowledge, less research effort has been devoted to elucidating the expression of TFEB in skeletal muscle, especially during or after exercise training (Mansueto et al. 2016). Thus, this study was designed to understand TFEB regulation in response to CCA over the time course of 7 days. Interestingly, we found that the CCA‐induced increase in TFEB protein levels preceded the time when muscle mitochondrial adaptations were fully attained in this exercise model. We also observed a significant positive correlation between TFEB and PGC‐1*α* protein expression levels, and this finding is supported by our previous studies, in which both loss‐ and gain‐of‐function of PGC‐1*α* led to parallel changes in TFEB expression in skeletal muscle (Vainshtein et al. 2015a). Collectively, these findings suggest the possibility that two transcription factors interact during skeletal muscle remodeling. However, more research is required on their codependence, since Mansueto et al. (2016) have documented a significant, PGC‐1*α*‐independent role for TFEB in mediating mitochondrial adaptations in skeletal muscle.

We also explored other lysosomal markers such as LAMP1 and Cathepsin D, both of which are also known to be regulated by TFEB (Spampanato et al. 2013; Vodicka et al. 2016). Although these markers were shown to be statistically increased by CCA during the time course of mitochondrial adaptations, we observed the most prominent protein expression changes by day 3, which seemed to occur prior to the largest increase in TFEB expression. It may be possible that those factors could be also regulated by other TFEB isoforms such as MITF and/or TFE3 in skeletal muscle. In this respect, Nezich et al. (2015) showed that starvation‐associated TFEB activation requires the presence of other TFEB homologues. In addition, it was suggested that LAMP 1 gene expression is significantly correlated with MITF gene expression, rather than with TFEB, in melanoma cells (Ploper et al. 2015). TFEB activity is also either dependent or independent on mTOR‐dependent phosphorylation, which regulates its translocation into the nucleus and further transcriptional activity related with lysosomal regulation. Indeed, muscle TFEB nuclear translocation was shown to be significantly upregulated following running exercise training (Mansueto et al. 2016), and future studies are needed to examine whether this TFEB translocation is also regulated in response to the current experimental model using nuclear and cytosolic fractionation.

As compared to other autophagic markers, chaperone‐mediated autophagy (CMA) has not received much attention for its role in regulating cellular quality control, particularly in skeletal muscle. Thus, we examined the levels of three key components of the process. HSC70, the constitutive isoform of chaperones, remained unaltered in response to CCA. However, LAMP2A was gradually increased in skeletal muscle during CCA‐induced adaptations. Similar results were reported by Tanaka et al. (2015), who found that 9 weeks of treadmill exercise training upregulated LAMP2A protein level in the adipose tissue of rats. In addition, MCOLN1 has been recently identified to play a significant role in autophagy through the regulation of TFEB translocation. Medina et al. (2015) showed that calcium is released from the lysosome through MCOLN1, and this regulates TFEB activity via calcineurin. In particular, the focal calcium pool around the lysosomal membrane appears to recruit and activate calcineurin to dephosphorylate TFEB and translocate it into the nucleus independent of mTOR complex. MCOLN1 has also been found to affect LAMP2A activity in the lysosomal storage diseases such as mucolipidosis IV (Venugopal et al. 2016). In this study, these two protein markers were both progressively increased in chronic contractile activity, supporting the possible functional linkage between MOCLN1 and LAMP2A. Taken together, our data suggest that CMA also plays an important role in CCA‐induced muscle adaptations, and it may be promising to further study the regulation of this process with a focus on TFEB translocation.

In summary, this study documents the chronology of autophagy protein changes during chronic exercise‐inducible muscle adaptations, and suggests that autophagy induction occurs coincident with mitochondrial biogenesis. Surprisingly, lysosomal adaptations appear to precede those related to autophagy and mitochondrial biogenesis, a phenotypic change which is likely useful to increase the capacity of muscle cells to accommodate increases in mitophagic and autophagic flux.

## Conflict of Interest

None declared.
